# Identification of Gene-Expression Signatures and Protein Markers for Breast Cancer Grading and Staging

**DOI:** 10.1371/journal.pone.0138213

**Published:** 2015-09-16

**Authors:** Fang Yao, Chi Zhang, Wei Du, Chao Liu, Ying Xu

**Affiliations:** 1 Key Laboratory for Symbolic Computation and Knowledge Engineering of the Ministry of Education, College of Computer Science and Technology, Jilin University, Changchun, China; 2 Computational Systems Biology Laboratory, Department of Biochemistry and Molecular Biology, and Institute of Bioinformatics, University of Georgia, Athens, United States of America; 3 Department of Oral and Maxillofacial Surgery, Shandong Provincial Hospital Affiliated to Shandong University, Jinan, China; 4 Jilin Teachers’ Institute of Engineering and Technology, Changchun, China; Shenzhen Institutes of Advanced Technology, CHINA

## Abstract

The grade of a cancer is a measure of the cancer's malignancy level, and the stage of a cancer refers to the size and the extent that the cancer has spread. Here we present a computational method for prediction of gene signatures and blood/urine protein markers for breast cancer grades and stages based on RNA-seq data, which are retrieved from the TCGA breast cancer dataset and cover 111 pairs of disease and matching adjacent noncancerous tissues with pathologists-assigned stages and grades. By applying a differential expression and an SVM-based classification approach, we found that 324 and 227 genes in cancer have their expression levels consistently up-regulated vs. their matching controls in a grade- and stage-dependent manner, respectively. By using these genes, we predicted a 9-gene panel as a gene signature for distinguishing poorly differentiated from moderately and well differentiated breast cancers, and a 19-gene panel as a gene signature for discriminating between the moderately and well differentiated breast cancers. Similarly, a 30-gene panel and a 21-gene panel are predicted as gene signatures for distinguishing advanced stage (stages III-IV) from early stage (stages I-II) cancer samples and for distinguishing stage II from stage I samples, respectively. We expect these gene panels can be used as gene-expression signatures for cancer grade and stage classification. In addition, of the 324 grade-dependent genes, 188 and 66 encode proteins that are predicted to be blood-secretory and urine-excretory, respectively; and of the 227 stage-dependent genes, 123 and 51 encode proteins predicted to be blood-secretory and urine-excretory, respectively. We anticipate that some combinations of these blood and urine proteins could serve as markers for monitoring breast cancer at specific grades and stages through blood and urine tests.

## Introduction

Breast cancer is a major threat to women's health, accounting for 22.9% of cancer cases in women [1]. According to the World Cancer Report [1], 458,503 cases of breast cancer–associated deaths worldwide were reported in 2008, which represents 13.7% of cancer-related deaths in women. It has been generally understood that breast cancer, probably other cancer types as well, of different stages and different grades require different treatment plans. For example, breast-conserving surgery plus radiation therapy is effective for most patients with early stage breast cancers [2] while systemic therapy are generally needed for advanced stage patients, such as hormone or chemo therapy, in addition to cancer-removal surgery and radiation. In addition, cancer grades are strongly associated with prognosis [3]. Specifically, more differentiated cancer grades tend to have more favorable prognosis. Clearly, correct classification of the grade and stage of a cancer has significant implications in determination of the treatment plan for a patient.

Cancer stages are used to reflect the size of a cancer tumor and its extent of invasion. It has been traditionally determined by cancer pathologists based on tumor size, nodal spread and metastasis [4]. In the recent past, molecular level information has been incorporated into the decision process of cancer staging, using markers such as alpha-fetoprotein and lactate dehydrogenase for determination of germ cell tumors [5]. A widely used system for cancer staging is that the cancer tissues are classed into four stages, namely I, II, III and IV, with a higher stage representing a more advanced cancer.

Cancer grading is a measure of the malignancy and aggressiveness independent of stage. Unlike staging, cancer grading has been predominantly done through visual inspection of the cell morphology and tissue structure [3], generally lacking in using molecular level information. Compared to stage determination, it is a less developed area in cancer classification. Currently there is no universal grading system for all cancer types, instead research communities of a few cancer types each have developed their own grading systems such as the one for breast cancer developed by Bloom and Richardson [6], the Gleason system for prostate cancer [7] and the Fuhrman method for kidney cancer [8]. While there are some differences in the detailed classification criteria, these grading systems generally classify cancer tissues to four grades: well differentiated (WD), moderately differentiated (MD), poorly differentiated (PD) and undifferentiated (UD).

A number of computational studies have been published on cancer staging and grading prediction based on transcriptomic data. For example, Cui et al have reported a 198-gene and a 10-gene panel for grading and staging prediction of gastric cancers, respectively [9]. For breast cancer, a grade index based on the expressions of 97 genes in cancer tissues was previously developed to classify patients with grade 2 tumors into two subgroups with high *versus* low risks of recurrence [10]. However, markers so developed have had only limited applications since tissue-based gene-expression data are generally not available for most patients [11, 12]. Hence, it is essential to extend tissue-based gene markers to markers that can be measured using blood or urine samples of patients [13, 14], the challenge of which is to predict reliably which of the overly expressed proteins in cancer tissues can be secreted into blood and further into urine.

In this study, we conducted a computational analysis tissue-based gene-expression data to identify possible gene signatures and blood/urine proteins markers for breast cancer grading and staging prediction. The following represents the unique contributions by this study, to the best of our knowledge: (1) RNA-seq-based gene-expression signatures for breast cancer grading and staging prediction; and (2) predicted potential marker proteins for cancer staging and grading that can be measured by using blood and urine samples. Clearly, this work represents only a pilot study for prediction of blood and urine marker proteins for breast cancer grading and staging. We expect that follow-up studies will demonstrate the feasibility of the predicted signature genes and protein markers.

## Results

### A. Identification of gene signatures for breast cancer

#### (1) Identification of gene groups whose expressions distinguish breast cancer from other cancers

Gene-expression data of 111 paired of breast cancer and adjacent control tissue samples were retrieved from the TCGA database [15], where each gene-expression dataset covers 20,501 human genes measured using RNA-seq. 5,562 differentially expressed genes between cancer and matching control tissues were identified using the following procedure: the expression levels of a gene in cancer show at least 2-fold change from the matching control tissues with the q-value < 0.05 to control the False Discovery Rate (FDR) (see Material and Methods). Among the 5,562 genes, 2,078 were up-regulated and 853 of them were found to be up-regulated in less than three out of 12 other cancer types that were examined in our study as references, hence making them as good candidates for breast cancer specific markers (see Material and Methods).

To predict gene signatures specific to breast cancer, we have searched for gene combinations among the 853 up-regulated genes in breast cancer, whose expression pattern can best distinguish breast cancer from other cancers and breast cancer from the control samples, using a support vector machine based feature elimination approach, named SVM-RFE (see Material and Methods). A 20-gene combination, {*COPA*, *GATA3*, *HDGF*, *LUM*, *SPINT2*, *STAT1*, *AEBP1*, *CALR*, *TRPS1*, *EPRS*, *ARL6IP1*, *EVL*, *RAD21*, *PKM2*, *CD9*, *NPNT*, *CLTC*, *CDH1*, *NAT1*, *SH3BGRL*}, has been identified, which can distinguish breast cancer from all other cancers, achieving a 94.3% average level of discrimination, and breast cancer from control samples with 99.9% accuracy. For detailed information of comparisons, we refer the reader to S1 Table.

#### (2) Identification of gene signatures for breast cancer grades

**Fig 1:**
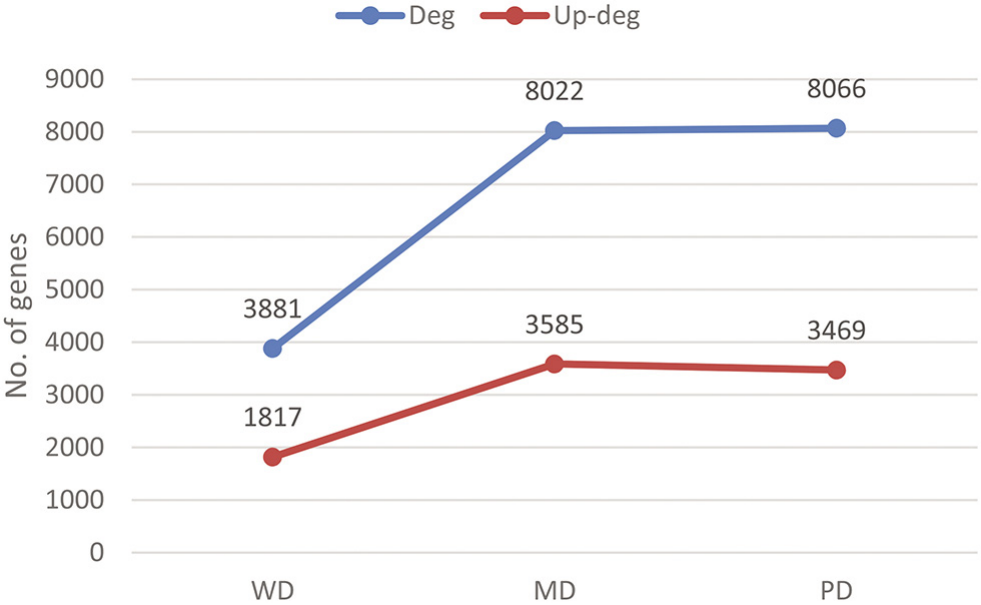
Cancer grades *versus* the number of differentially expressed genes. The blue and red lines are for the numbers of differentially expressed and up-regulated genes, respectively, across the three grades considered.

Out of the 111 cancer samples used in this study, 11, 40 and 28 are well, moderately and poorly differentiated, respectively, with the remaining having no grade information provided. 3,881 genes are found to be differentially expressed between the WD and the matching control tissues, with 1,817 genes being up-regulated. 8,022 genes are differentially expressed between the MD tissues and matching controls, with 3,585 up-regulated; and 8,066 genes are differentially expressed between the PD tissues and the controls, with 3,469 up-regulated. We noted that there is a clear trend that the number of differentially expressed genes increases as the grade going from more to less differentiated, as shown in Fig 1. This observation is in agreement with our knowledge that less differentiated cancers tend to be more malignant.

**Fig 2:**
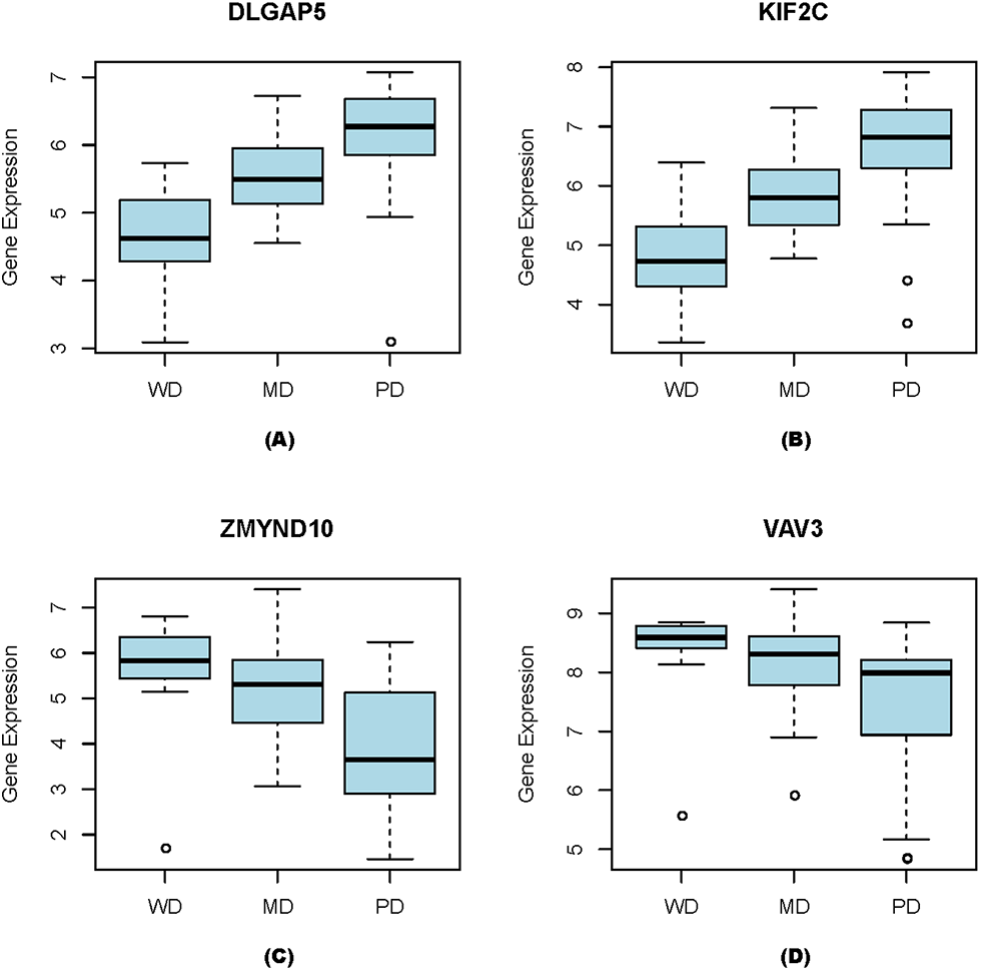
Correlation between gene-expression levels and three grades of breast cancer. (A) DLGAP5, (B) KIF2C, (C) ZMYND10, and (D) VAV3.

We have checked if some genes have their expression-level changes correlate with the cancer grades. Significant correlation between the level of up-regulation and the three cancer grades WD-MD-PD has been identified of 324 up-regulated genes, as detailed in S2 Table, where the statistical correlation is assessed using the Spearman correlation coefficient and the Mann Whitney test (see Material and Methods). Fig 2 shows four such genes, (*DLGAP5*, *KIF2C*, *ZMYND10*, *and VAV3*), with their overexpression levels positively or negatively correlate with the breast-cancer grades. It is noteworthy that *DLGAP5* has been found that its silencing suppresses tumorigenicity and inhibits cellular proliferation in cancer cells [16]. *KIF2C* has been reported that its overexpression involves in breast carcinogenesis [17]. *ZMYND10* is a tumor suppressor gene in neuroblastoma [18]. *VAV3* has been reported to serve as an oncogene and its overexpression is associated with poor prognosis of a breast cancer [19].

A function enrichment analysis of the 324 genes has been conducted by using a hypergeometric test against 2,801 pathways covering the GO terms, canonical pathways from Msigdb [20] and our manually collected gene sets [20, 21] (see Material and Methods). 103 pathways are significantly enriched by these genes with a significance level < 0.001. The top ten most significantly enriched gene sets/pathways, along with significance values, are shown in Table 1, with the complete list of the enriched pathways provided in S3 Table. Note that around 85% (88/103) of the enriched pathways are cell cycle, DNA replication and damage repair, and cell proliferation regulation related, suggesting the most significant difference among the breast cancers of different grades is the tumors’ cell proliferation rate.

**Table 1:** The top ten most significantly enriched pathways by the 324 genes.

Pathway name	Gene count	Size of gene set	P value
REACTOME_CELL_CYCLE_MITOTIC	54	325	1.60E-27
REACTOME_CELL_CYCLE	60	421	4.41E-27
CELL_CYCLE_PROCESS	43	193	6.77E-27
REACTOME_DNA_REPLICATION	41	192	3.58E-25
CELL_CYCLE_PHASE	38	170	3.95E-24
MITOTIC_CELL_CYCLE	35	153	1.05E-22
M_PHASE	31	114	3.02E-22
CELL_CYCLE_GO_0007049	46	315	6.74E-22
REACTOME_MITOTIC_M_G1_PHASES	35	172	2.80E-21
M_PHASE_OF_MITOTIC_CELL_CYCLE	25	85	4.95E-19

Here, the gene count denotes the number of the 324 genes observed in each pathway; the size of a gene set is the total number of genes in the gene set or pathway, and the p-value is the significance level of the enrichment calculated by the hypergeometric test.

We have then searched among the 324 genes to find combinations among them whose expression patterns can distinguish among different cancer grades. A 9-gene combination, (*FGD3*, *CENPI*, *AURKB*, *DEPDC1B*, *FAM83D*, *NCAPH*, *TNFRSF18*, *FCGR1A*, *DEPDC1*), has been identified, whose expression pattern can distinguish the PD group from the MD and WD groups with 96.3% classification accuracy (94.5% sensitivity and 97.3% specificity). Similarly, a 19-gene combination, (*EPR1*, *CREB3L1*, *BGN*, *CXCL10*, *UBE2S*, *INHBA*, *CEP55*, *BUB1*, *KIFC1*, *CDC45*, *SPATA17*, *CA12*, *CILP2*, *PTTG1*, *ADAMTS14*, *CLEC5A*, *FGD3*, *TNFRSF18*, *NEIL3*), has been identified that could distinguish the MD group from the WD group with 94.2% classification accuracy (95.0% sensitivity and 92.2% specificity).

#### (3) Identification of gene signatures for breast cancer stages

**Fig 3:**
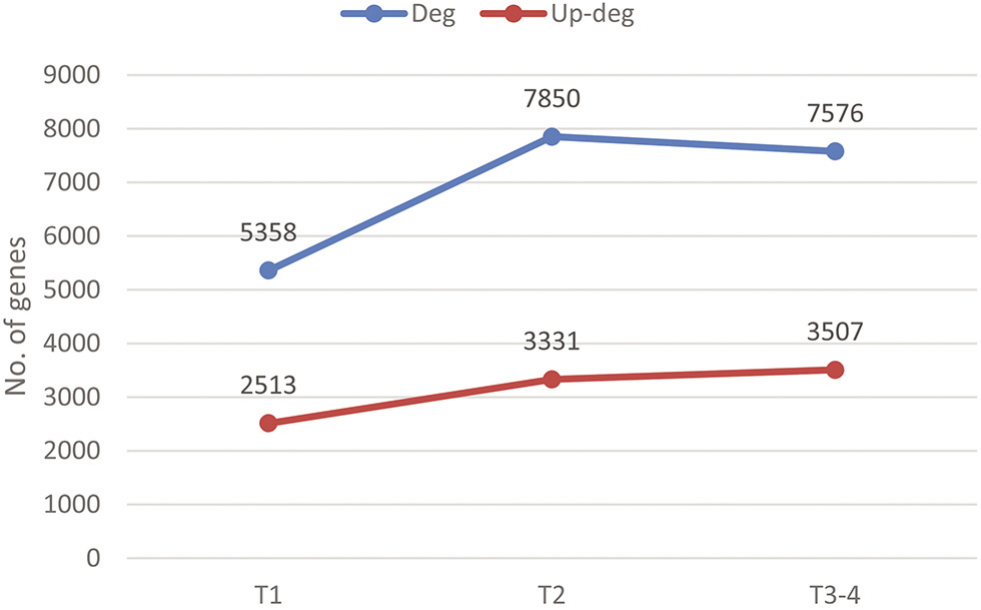
Cancer stages *versus* the number of differentially expressed genes. The blue and red lines are for the numbers of differentially expressed and up-regulated genes, respectively, across the three stages.

A similar approach is used to identify stage-dependent gene combinations. Out of the 111 cancers samples, 12 are in stage I (T1), 47 in stage II (T2), 19 in stage III (T3) and 1 in stage IV (T4) with the remaining not having such stage information. Considering that stage IV has only one sample, we combined samples in stages III and IV into one stage T3-4. We have observed: 5,358 genes are differentially expressed in T1 samples *versus* controls, with 2,513 up-regulated; 7,850 are differentially expressed in T2 samples *versus* controls, with 3,331 up-regulated; and 7,576 are differentially expressed in T3-4 samples *versus* controls, with 3,507 up-regulated. All this information is summarized in Fig 3, which shows an upward trend in the number of differentially expressed genes from early to more advanced stages, similar to that for grade-dependent genes.

**Fig 4:**
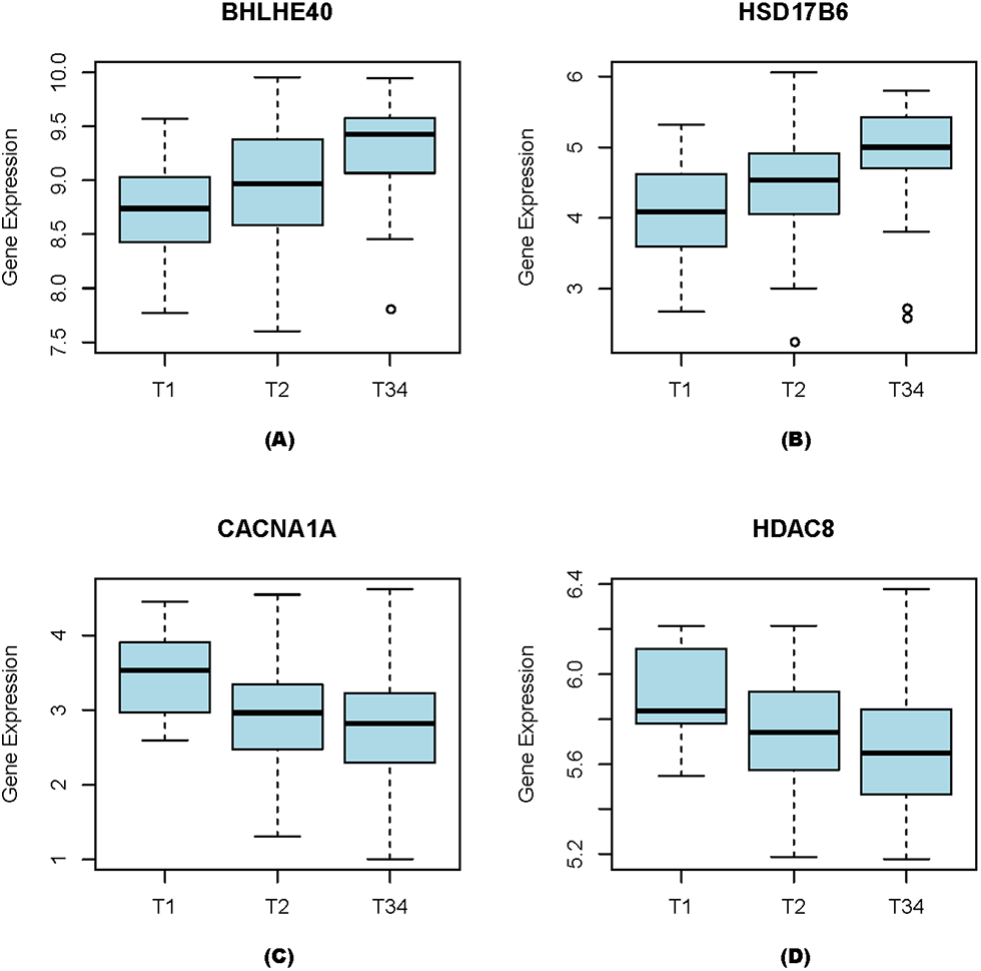
Correlation between gene-expression levels and three grades of breast cancer. (A) BHLHE40, (B) HSD17B6, (C) CACNA1A, and (D) HDAC8.

We have also checked if some genes may have their expression-level changes correlate with cancer stages. Overall, 227 up-regulated genes are found to have their expression levels correlate with cancer progression from T1 through T3-4, as detailed in S4 Table. Fig 4 shows four such examples, (*BHLHE40*, *HSD17B6*, *CACNA1A*, *HDAC8*), each with either positive or negative correlation with the stage progression. Among them, *BHLHE40* has been reported to correlate with the increased malignancy potential and invasiveness in breast cancer [22]. *HSD17B6* is known to be a key enzyme that can catalyze the conversion of 3α-diol to DHT in prostate cancer [23]. *CACNA1A* is predicted to be a tumor suppressor gene in lung cancer [24]. *HDAC8* has been reported to link to the dysregulated expression or interaction with transcription factors critical to tumorigenesis [25].

Pathway enrichment analysis has also been carried out on the 227 stage-dependent genes. 59 pathways have been found to be significantly enriched by these genes, including carbohydrates metabolism, ion metabolism and homeostasis, mRNA metabolism, apoptosis, ER stress, ABC transporters, protein binding, response to acidosis plus a few signaling pathways. A few of the enriched pathways are listed in Table 2 while the complete set of the enriched pathways are given in S5 Table. Unlike grade-dependent genes, the stage-dependent genes enrich pathways with more diverse functions, but predominantly metabolism or homeostasis related. Hence we infer the key changes as a breast cancer advances are related to micro-environmental factors and responses, which is consistent with our previous result that multiple cancer types (including breast cancer) continuously alter their micro-environments, including the levels of hypoxia and oxidative stress, as a cancer advances [21, 26].

**Table 2:** Selected enriched pathways.

Pathway name	Gene counts	Size of the pathway	P value
TRANSCRIPTION_COACTIVATOR_ACTIVITY	10	123	3.10E-05
BIOCARTA_CHEMICAL_PATHWAY	5	22	6.74E-05
INTRACELLULAR_ORGANELLE_PART	32	1192	6.94E-05
REACTOME_APOPTOSIS	10	148	7.88E-05
REACTOME_GLYCOSAMINOGLYCAN_METABOLISM	9	111	0.000136
REACTOME_INTRINSIC_PATHWAY_FOR_APOPTOSIS	5	30	0.000153
KEGG_LYSOSOME	9	121	0.000195
REACTOME_METABOLISM_OF_CARBOHYDRATES	11	247	0.000222
ION_TRANSPORT	10	185	0.000235
REACTOME_DEVELOPMENTAL_BIOLOGY	13	396	0.000248
STEROID_HORMONE_RECEPTOR_BINDING	3	10	0.000298
CELL_DEVELOPMENT	23	577	0.000337
KEGG_PORPHYRIN_AND_CHLOROPHYLL_METABOLISM	5	41	0.000352
UNFOLDED_PROTEIN_BINDING	5	42	0.000375
KEGG_ABC_TRANSPORTERS	5	44	0.000424
CATION_TRANSPORT	9	147	0.000435
GLUCOSAMINE_METABOLIC_PROCESS	3	13	0.000468

In the table, the gene count denotes the number of stage-dependent genes observed in each pathway; the size of a gene set is the total number of genes in each gene set or pathway; and the p-value is the significance level of the enrichment calculated using a hypergeometric test.

We have searched among the 227 up-regulated genes to find combinations among them whose expression patterns can distinguish among different cancer stages. Using an analysis similar to that for grade-dependent genes, we have identified a 30-gene combination whose expression pattern can best distinguish advanced stage (T3-4) from early stage (T1+T2) breast cancers, with an 99.9% classification accuracy (99.5% sensitivity and 100% specificity). The 30 genes are *OR6K3*, *RMND5B*, *LPCAT3*, *FRRS1*, *LOC728554*, *RFX5*, *JAKMIP1*, *CLGN*, *NDST3*, *GPR6*, *RIPK3*, *C2CD4A*, *PCDHA8*, *LENEP*, *CGA*, *GABRD*, *DLX1*, *GPR39*, *C1orf227*, *KLF1*, *ANXA10*, *EIF3C*, *UQCRQ*, *MAPKAPK3*, *SH3BP5L*, *TCTEX1D2*, *TCL1A*, *IFT122*, *RAET1L* and *ABCC13*. Similarly we identified a 21-gene combination, (*C1orf141*, *DNAJC15*, *FIG4*, *LAPTM4B*, *HRASLS2*, *SEMA4A*, *SLC25A24*, *POTEH*, *SLC4A2*, *CLEC4C*, *MRPS21*, *AP3S1*, *CLDN6*, *CST6*, *HHLA2*, *GPR6*, *ABCC13*, *AZIN1*, *OTX2*, *MPP2*, *CAPZB*), whose expression pattern can distinguish the T2 tissues from the T1 tissues, with 98.0% classification accuracy (99.7% sensitivity and 91.3% specificity).

#### (4) Validation of the identified gene markers

Five breast cancer microarray datasets, with either grade or stage information, in the GEO database are analyzed to validate the gene markers predicted in the previous sections, to demonstrate that the predicted markers are stable on different datasets collected by different groups. The detailed information of the datasets are given in S9 Table.

For the predicted gene markers, we have examined their expression patterns with respect to stages and grades, respectively, in the test microarray datasets by using the Mann Whitney test with significance level 0.05 as the cutoff. Considering that there could be an intrinsic difference between RNA-seq and microarray data, we have first tested the stability of the marker genes predicted using the RNA-seq data on the matching microarray data over the same set of tissue samples, which are made available in the TCGA database. 81.3% (234/288) of the grade-dependent marker genes and 44.7% (83/183) stage-dependent marker genes passed the above statistical test, suggesting the level of the intrinsic difference between RNA-seq and microarray data based predictions.

We then examined the predicted grade and stage-dependent marker genes against three microarray data sets retrieved from the GEO database with grade and stage information available, respectively. We noted that 92.8% (284/306) of the grade-dependent genes passed the statistical test in at least one of the three data sets with grade information and the average validation rate across the three datasets is 76.4%. Meanwhile, 42.7% (89/208) of the stage dependent marker genes passed the test in at least one out of three datasets with stage information and the average validation rate across the three data sets is 21.67%. Detailed statistics of the validations are listed in S2 and S4 Tables. It is worth noting that the percentage of the validated marker genes is much higher than the expected discovery rate, i.e. 5%, suggesting the overall reliability of the identified gene markers.

We have also assessed the discerning power of each predicted gene combination for breast cancer staging and grading on the microarray datasets. On the TCGA microarray dataset (matching the RNA-seq data), the 9-gene combination for distinguishing the PD group from the MD and WD groups achieves 87.08% prediction accuracy with 79.83% sensitivity and 89.63% specificity; the 19-gene combination for MD versus WD classification achieves 83.13% prediction accuracy with 91.83% sensitivity and 50.00% specificity. For cancer staging, the 21-gene combination for classification between the T1 and T2 groups and the T3-4 group achieves 76.38% prediction accuracy with 48.00% sensitivity and 85.95% specificity; and the 18-gene combination for T1 versus T2 classification achieves 80.20% prediction accuracy with 89.33% sensitivity and 54.00% specificity. Detailed statistics are shown in Table 3.

**Table 3:** The prediction accuracy of the identified gene combinations on TCGA microarray data.

	Number of genes	Accuracy	Sensitivity	Specificity
PD vs.MD,WD	9-gene	87.08%	79.83%	89.63%
MD vs. WD	16-gene	83.13%	91.83%	50.00%
T34 vs.T1,T2	21-gene	76.38%	48.00%	85.95%
T2 vs. T1	18-gene	80.20%	89.33%	54.00%

On the other three microarray datasets, the gene combination for distinguishing the PD group from the MD and WD groups achieves 77.04% prediction accuracy with 80.03% sensitivity and 72.03% specificity, and the gene combination for discerning the MD group from the WD group achieves 71.73% prediction accuracy with 77.72% sensitivity and 56.67% specificity. Similarly, the gene combination for distinguishing stages T1 and T2 from T3-4 achieves 59.34% accuracy with 32.72% sensitivity and 63.13% specificity; and the gene combination for T1 and T2 discrimination achieves 69.60% accuracy with 81.72% sensitivity and 23.80% specificity on average.

It is worth noting that a partial reason for the reduced performance level of our predicted markers on the microarray data is that some selected genes based on the RNA-seq data are missed in the microarray data and some genes expression levels may be not accurately reflected in microarray data due to the intrinsic limitations of the technique [27].

We have also conducted SVM-RFE-based classifier training directly using microarray data and the 324 grade and 227 stage-dependent genes identified based on RNA-seq data. Across the board, gene combinations were identified to achieve better than 90% prediction accuracy with at least 90% sensitivity and 90% specificity for each of the prediction tasks discussed above. Detailed performance statistics by these predicted gene combinations are given in S10 Table.

### B. Prediction of protein biomarkers for breast cancer in blood and urine

#### (1) Prediction of protein biomarkers for breast cancer

Overall, 853 genes were found to be up-regulated in breast cancer *versus* control tissues, which are up-regulated in at most three other cancer types out of the 12 we have examined as controls (see Material and Methods), hence making them as potential candidates for identification of unique gene combinations as done above. We then analyzed which of their protein products can be secreted into circulation, using a predictor developed previously by our lab [28]. 415 of these genes are predicted to encode blood secretory proteins, hence making them as potential blood biomarkers for breast cancer detection through blood test. Some of these proteins have been previously reported to be breast cancer related biomarkers, such as *C4a* [29], *HER2* [30], *CA15-3* [31], *alpha-1-antichymotrypsin* [32] and *alpha-1B-glycoprotein* [33]. Overall our extensive literature survey found that 5 out of the 415 proteins have been reported to be found in blood circulation, giving rise to a p-value 0.045 if the 415 proteins are selected by chance and hence providing an overall confidence level of our prediction.

Similarly, we have applied our prediction tool for urine excretory proteins [34] for the 853 genes identified in Section A(1), and predicted that 176 can have their protein products excreted into urine. As of now, no proteins have been reported to be urinary biomarkers for breast cancer, to the best of our knowledge.

To further narrow down the candidate protein biomarkers in blood and urine for experimental validation from the 415 and 176 genes, respectively, we have considered combinations of some of these genes in a fashion similar to the analysis in the previous section, to suggest the most informative combinations as potential blood and urine biomarkers for breast cancer detection. At the end, we found one 36-gene combination from the 415 genes, whose protein concentration level could be the most distinguishing between breast and other cancers: *MAPKAPK2*, *PARP1*, *CCT3*, *VAV3*, *AEBP1*, *KDM5B*, *NPNT*, *TMED3*, *NEBL*, *STAT1*, *POGK*, *ATP2A3*, *FKBP4*, *ABHD2*, *EFNA1*, *PRSS8*, *CALR*, *LUM*, *MAZ*, *PDXDC1*, *SPINT1*, *REPS2*, *CREB3L4*, *PGK1*, *KIAA1522*, *SIPA1L3*, *GBP5*, *TTLL12*, *ZNF217*, *ARNT2*, *FOXRED2*, *ALDH18A1*, *RSAD2*, *TGFB3*, *PCK2* and *SERPINA3*, with detailed prediction data given in S6 Table. We also predicted a 15-gene combination from the 176 genes, whose urinary proteins may serve as a good urinary biomarker for breast cancer against other cancers: *B4GALT3*, *RAB31*, *EFNA1*, *NPNT*, *SEMA4A*, *H2AFZ*, *SMARCA4*, *H2AFY*, *NSF*, *HIST1H2AC*, *CDH1*, *H3F3A*, *CLTC*, *EZR* and *HLA-DQA2*, with detailed prediction information given in S6 Table.

#### (2) Prediction of protein biomarkers for breast cancer grades

In a very similar fashion, we have predicted blood biomarkers for highly *versus* lowly differentiated breast cancers using the 324 up-regulated grade dependent genes. 188 of the 324 proteins were predicted to be blood secretory and 66 were urine excretory. These proteins could be used as potential blood and urine biomarkers for breast cancer grades, respectively. Some of these proteins have been reported to be breast cancer related markers, such as *Ki-67* (*MIB-1*) [31] and *CA15-3* [31] being blood secretory protein markers for breast cancer, and *C-telopeptide* of collagen type I [35], which contains two chains, being urine excretory protein markers. Overall at least 2 out of the 66 proteins have been found in urine, giving rise to a p-value 0.0004 if the 66 proteins are selected by chance and hence providing an overall confidence level of our prediction.

We have also examined if some combinations of the 188 genes’ protein products may have high distinguishing power among breast cancers of different grades. A 19-gene combination is identified that can best distinguish the PD group from the MD and WD groups with a 93.6% classification accuracy based on gene-expression data. Similarly, a 17-gene combination is predicted to have strong discriminating power between the MD group and the WD group, with a 93.3% classification accuracy.

Similarly, we have examined if some combinations of the 66 urine excretory genes’ proteins have high distinguishing power among breast cancers of different grades. A 15-gene combination has been found to well distinguish the PD group from the MD and WD groups, with a 92.8% classification accuracy based on gene-expression data. And a 19-gene combination is predicted to distinguish the MD group from the WD group, with a 83.8% classification accuracy. The detailed gene lists of these blood and urine gene combinations are given in S7 Table.

#### (3) Prediction of protein biomarkers for breast cancer stages

Using a similar prediction procedure to that in the above section, a 23-gene combination is predicted to be the best distinguisher between the T3-4 and the T2-T1 group, with a 99.4% classification accuracy based on gene-expression data. And a 23-gene combination is predicted to have the best discerning power to distinguish between the T2 group and the T1 group, with a 98.1% classification accuracy.

Similarly for urine secretory gene panels, a 19-gene combination is predicted to best distinguish the T3-4 group from the T2-T1 groups, with a 87.6% classification accuracy. And a 25-gene combination is predicted to best distinguish the T2 from the T1 group, with a 97.2% classification accuracy. The detailed gene lists of these stage biomarkers are given in S8 Table.

## Discussion and Conclusion

Reliable prediction of a cancer’s grade and stage is very important as it can provide useful information for cancer mechanism studies as well as for selection of the most appropriate treatment plans. In this study, we used our in-house computational approaches to have predicted reliable gene signatures and protein markers for breast cancer detection and their grades and stages by using 111 pairs of breast cancer and matching control tissues. In order to identify the most reliable markers, we specifically applied the non-parametric Mann Whitney test with relatively lower sensitivity but higher specificity compared to other parametric tests for differential gene expressions [36]. In addition, an SVM-RFE based approach is used to select a combination of genes that can best discriminate between two specific groups of cancer tissues such as cancers in two different grades or stages [37]. Such a method has been widely used in analyzing high-dimensional biological data for feature selection. Our experience has been that an SVM with a linear kernel tend to achieve the desired prediction accuracy without a major concern about over-fitting.

We noted that among the predicted marker genes, breast cancer specific markers, especially the 20 identified by SVM-RFE, tend to be cancer associated genes such as oncogene CLTC and tumor suppressor genes CDH1 and GATA3 while PKM2, STAT1, EPRS, HDGF, LUM, SPINT2, TRPS1, EVL, RAD21, NPNT, NAT1, whose gene expression level changes have been reported as breast cancer associated [20, 38–49]. Among the two classes of markers, most of the grade markers are cell-proliferation related while the stage markers relate to more diverse biological functions such as metabolisms, apoptosis and cancer micro-environmental stresses, hence revealing useful information about molecular level differences among breast cancers of different grades as well as of different stages.

By using our in-house software, we predicted the possible blood and urine protein markers based on the predicted uniquely over-expressed genes in breast cancer. Such information could provide useful targets for guided search for protein markers in blood and/or urine for breast cancer detection and/or classification through blood or urine tests. We fully expect follow-up studies will demonstrate the feasibility of the predicted signature genes and protein markers.

## Material and Methods

### 1. RNA-seq data

RNA-seq data of breast cancer and 12 other cancer types, namely bladder carcinoma, colon adenocarcinoma, head and neck squamous cell carcinoma, kidney chromophobe, kidney renal clear cell carcinoma, kidney renal papillary cell carcinoma, liver hepatocellular carcinoma, lung adenocarcinoma, lung squamous cell carcinoma, prostate adenocarcinoma, rectum adenocarcinoma and thyroid carcinoma were downloaded from the TCGA database [50], all of which were measured by Illumina HiSeq platform and normalized by the RSEM method [51]. Each of the selected cancer types has at least 10 cancer and matching control samples. It is worth noting that the RNA-seq data of these 13 cancer types are measured and normalized by the same procedure, making comparisons among differential gene-expressions across different cancer types feasible. Detailed cancer grade and stage information of each sample are also accessed from TCGA.

In addition to the RNA-seq data, the matching microarray data collected on the same breast cancer and control samples are also retrieved from TCGA. In addition, five microarray data sets collected on independent collections of tissue samples are retrieved from the GEO database and analyzed to validate our RNA-seq data based marker predictions. The detailed information of all these data is listed in S9 Table.

### 2. Identification of differentially expressed genes

Our previous study has revealed that the normalized microarray or RNAseq gene expression profile through multiple cancer samples may follow mixed distributions with multiple peaks due to possible intra-tumor heterogeneity or disease sub-types as shown in S1 Fig [52]. Hence for the datasets with paired samples of cancer and adjacent control tissues, the non-parametric Wilcoxon signed-rank test [53] was applied to identify genes that are differentially expressed in cancer *versus* control samples. Specifically, our null hypothesis *H*_0_ is that a gene is not differentially expressed in cancer *versus* the control samples; rejection of this hypothesis means that the gene is differentially expressed in cancer versus the controls. Let *C*_*i*_ and *N*_*i*_ be a gene’s expression level in the *i* ^−*th*^ cancer and the matching control tissues, *i* = 1,…,*N* and *N* being the number of paired samples. We calculate |*C*_*i*_ − *N*_*i*_| and sgn(*C*_*i*_ − *N*_*i*_), with $\mathrm{sgn}\left(x\right)=\{\begin{array}{c} 1\;if\;x>0,\\ 0\;if\;x=0,\\ -1\;if\;x<0. \end{array}$. We exclude all tissue pairs with |*C*_*i*_ − *N*_*i*_| = 0 in our gene-expression data analyses. Let *N*_*r*_ be the remaining sample size, and sort the *N*_*r*_ tissue pairs in the increasing order of the |*N*_*i*_ − *C*_*i*_| values. We then give each pair a rank, numbered contiguously, consistent with the relative positions in the sorted list of paired tissues, i.e., with the first pair of tissues having rank 1 and tissues with the same |*N*_*i*_ − *C*_*i*_| value having the same rank, overall represented by *R*_*i*_. We calculate the test statistic *W* using $W=\left|\sum_{i=1}^{N_{r}}\left[\mathrm{sgn}\left(C_{i}-N_{i}\right)\cdot R_{i}\right]\right|$. For *N*_*r*_ ≥ 10, a *z-score* is calculated as $z=\frac{W-0.5}{\sigma_{W}}$ and $\sigma_{W}=\sqrt{\frac{N_{r}\left(N_{r}+1\right)\left(2N_{r}+1\right)}{6}}$. If *z* > *z*_*critical*_, the null hypothesis *H*_0_ is rejected. For *N*_*r*_ < 10, *W* is compared to a critical value from a reference table. If $W\geq W_{critical,N_{r}}$, *H*_0_ is rejected, i.e. we consider the gene as differentially expressed.

For each cancer type, significantly differential expression is determined by the False Discovery Rate (FDR) < 0.05 [54] and the fold-change in the expression levels in cancer *versus* the matching control be larger than 2.0. For breast cancer grades and stages, significantly differential expression is determined by FDR < 0.05 and the fold-change > 1.5.

### 3. Identification of genes whose differential expressions correlate with cancer grades and stages

Spearman correlation coefficient [55] was used to assess the level of correlation between the average gene expression and the sample stage or grade for identifying genes whose expression change go up or down strictly monotonically with respect to stages or grades. The Mann Whitney test is then applied to identify the differentially expressed genes among the different stages or grades with p < 0.05 as the cutoff for the significance level.

### 4. Pathway enrichment analysis

Pathway enrichment is assessed using a hypergeometric test against 2,801 gene sets covering pathways in the KEGG [56], Biocarta [57], Reactome databases [58] and the GO terms [59] collected from Msigdb and our manually collected cancer micro-environmental stress associated gene sets [20, 21]. In order to control the false discovery rate, we used the statistical significance p = 0.001 as the cutoff for a pathway enrichment test.

### 5. Prediction of gene signatures for cancers in a specific grade or stage

A support vector machine (SVM)-based recursive feature elimination approach was applied to predict gene signatures for each breast cancer grade as well as stage. A linear SVM was used for training our classifier [60, 61]. It constructs a hyper-plane to separate two different classes of feature vectors to achieve a maximum margin. This hyper-plane is constructed by finding a vector w and a variable b that minimize ‖*w*‖^2^, which satisfies the conditions *y*_*i*_(*w*∙*x*_*i*_ + *b*) ≥ 1, where *x*_*i*_ is a feature vector, *y*_*i*_ is 1 or -1, representing the class to which the point *x*_*i*_ belongs. Gene signatures of each training set were selected by using the recursive feature elimination procedure [62]. The overall accuracy of a trained classifier was evaluated using the 5-fold cross-validation and leave one out method [63].

### 6. Prediction of genes that encode blood-secretory and urine-excretory proteins

All up-regulated genes in breast cancer were analyzed for predicting if their protein products are blood-secretory, using a program developed by our lab [28], and urine-excretory, using a program developed also by our lab [34].

The basic idea of each algorithm is as follows. Human proteins known to be blood secretory (urine excretory), according to the published data, are selected to form a positive training set and proteins, known to be not blood secretory (urine excretory), are selected to form a negative training set. A list of features related to protein sequence and structures were examined and those found to have discerning power between the positive and the negative training data were selected. A (SVM)-based classifier for blood secretory (urine excretory) proteins. Both programs have been systematically assessed against large datasets, having achieved high-level prediction accuracy in both cases.

## Supporting Information

S1 Table List of gene signatures, whose expression pattern can best distinguish breast cancer from other cancers, and breast cancer from control samples. (XLSX)

S2 Table List of the 324 breast cancer grade associated genes. In the table, the column p value and sign represent the p value of the differential expression and up (“+”) or down (“-”) regulation between the two labeled classes, respectively. The TCGA RNAseq data analysis results and microarray validation results are colored in green and yellow respectively while “IS” represents insignificant. Blank elements in the validation columns suggest the genes are non-differentially expressed in the RNAseq data.
(XLSX)

S3 Table The 103 identified pathways that are significantly enriched by the 324 up-regulated grade-associated genes. Non-cell proliferation associated pathways are yellow highlighted.
(XLSX)

S4 Table List of the 227 breast cancer stage associated genes. In the table, the column p value and sign represent the p value of the differential expression and up (“+”) or down (“-”) regulation between the two labeled classes, respectively. The TCGA RNAseq data analysis results and microarray validation results are colored in green and yellow respectively while “IS” represents insignificant. Blank elements in the validation columns suggest the genes are non-differentially expressed in the RNAseq data.
(XLSX)

S5 Table The 59 identified pathways that are significantly enriched by the 227 up-regulated stage-associated genes. (XLSX)

S6 Table Gene lists, which corresponding proteins may serve as potential blood and urine biomarkers for breast cancer. (XLSX)

S7 Table Gene lists, whose corresponding proteins severs as potential blood and urine biomarkers for breast cancer grades. (XLSX)

S8 Table Gene lists, whose corresponding proteins severs as potential blood and urine biomarkers for breast cancer stages. (XLSX)

S9 Table Detailed information of the analyzed data. (XLSX)

S10 Table Statistics of the validation of SVM-RFE predicted grade and stage classifiers. (XLSX)

S1 Fig Histogram of four selected gene expression profiles in TCGA breast cancer data. In each figure, the gene expression prolife (RSEM value) of TCGA breast cancer samples and normal breast samples are colored by blue and pink, respectively. The expression profile cancer samples are fitted by mixed Gaussain distributions. The red, blue and green curves represent the density function of the fitted mixed Gaussain distributions (weighted by sample size).
(PDF)
