# Supplementary figures and images for: Identification of Gene-Expression Signatures and Protein Markers for Breast Cancer Grading and Staging
Source: PLoS One. 2015 Sep 16;10(9):e0138213. doi: 10.1371/journal.pone.0138213 (PMC4573873; doi:10.1371/journal.pone.0138213)

**CDC45 RSEM**

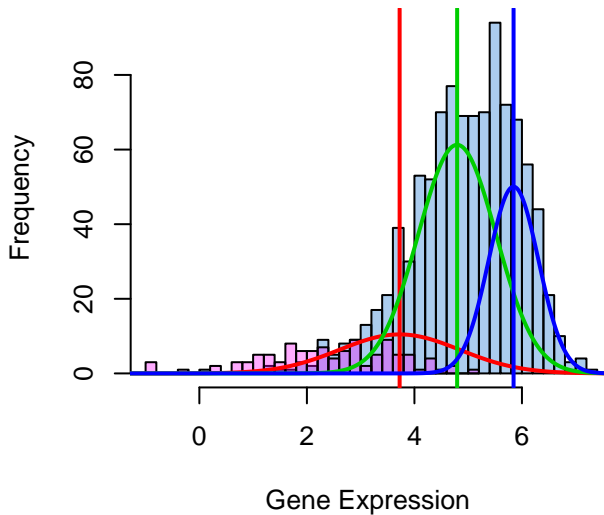

**DNALI1 RSEM**

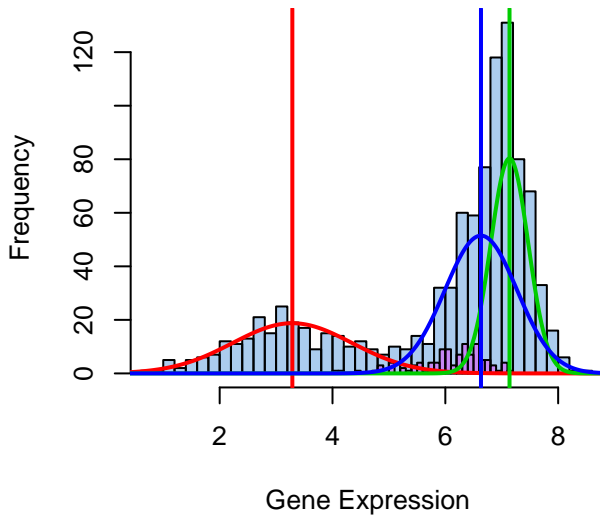

**PREX1 RSEM**

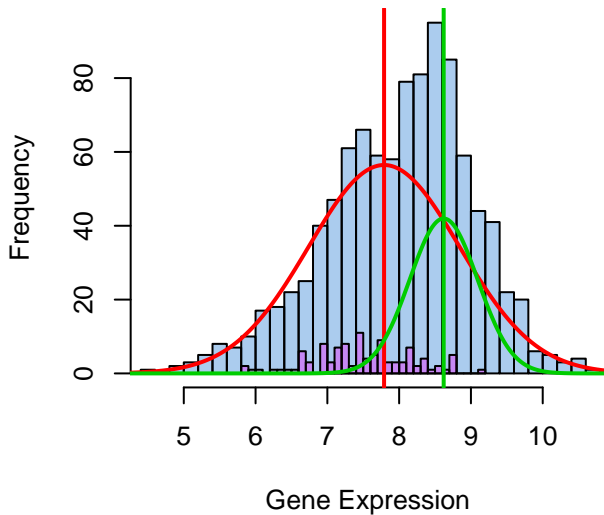

**ESR1 RSEM**

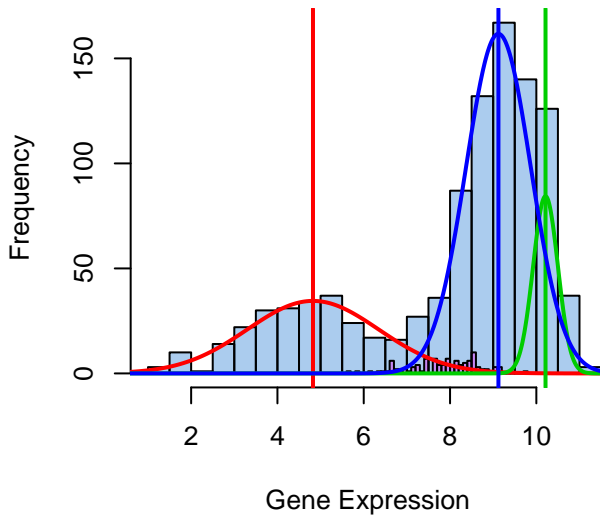

Supplement: S1 Fig — In each figure, the gene expression prolife (RSEM value) of TCGA breast cancer samples and normal breast samples are colored by blue and pink, respectively. The expression profile cancer samples are fitted by mixed Gaussain distributions. The red, blue and green curves represent the density function of the fitted mixed Gaussain distributions (weighted by sample size). (PDF) [file pone.0138213.s011.pdf]
